# Enhancing Urban Health Through a Community of Practice to Promote Active Lifestyle in a Population with Chronic Diseases: The +ACTIU Project

**DOI:** 10.3390/ijerph22121833

**Published:** 2025-12-07

**Authors:** Mercedes Gil-Lespinard, Olga Canet-Vélez, Júlia Ollé-Gonzalez, Assumpta Casas-Camí, Celia García Albertos, Marta Rofín Serrà, Jordi Vilaró Casamitjana, Paula Jakszyn

**Affiliations:** 1 Unit of Nutrition and Cancer, Cancer Epidemiology Research Programme, Catalan Institute of Oncology—Bellvitge Biomedical Research Institute, 08908 Barcelona, Spain; mgill@idibell.cat; 2 Global Health, Gender and Society (GHenderS), Blanquerna School of Health Sciences, Ramon Llull University, 08025 Barcelona, Spain; olgacv@blanquerna.url.edu (O.C.-V.); juliaog@blanquerna.url.edu (J.O.-G.); aumptacc@blanquerna.url.edu (A.C.-C.); 3 Healthy Cities SL, Bax Innovation Consulting SL, 08013 Barcelona, Spain; c.garcia@baxcompany.com (C.G.A.); m.rofin@baxcompany.com (M.R.S.); 4 Global Research on Wellbeing (GRoW), Blanquerna School of Health Sciences, Ramon Llull University, 08025 Barcelona, Spain; jordivc@blanquerna.url.edu

**Keywords:** urban health, sedentary behaviour, physical activity, non-communicable diseases, community

## Abstract

Urban environments play an important influence in influencing healthy lifestyles and reducing sedentary behaviour (SB), particularly as facilitators of physical activity (PA). Urban spaces often do not support healthy lifestyles. A Community of Practice (CoP) could be a valuable strategy for co-designing proposals to enhance healthy and active urban environments. We aimed to develop strategies through a CoP to promote PA and reduce SB in the urban setting of a multicultural population based in the Barcelona Metropolitan Area, with a particular focus on people with chronic diseases. A three-session CoP involving 25 participants (community members with chronic conditions, health professionals, urban planners, and local authorities) was implemented as a participatory research approach to identify barriers and facilitators to PA and co-design feasible urban improvement proposals. Ethical approval was obtained from Bellvitge University Hospital’s Ethics Committee. Participants provided informed consent and image release forms. Participants highlighted the importance of accessible, adaptable, and interconnected urban spaces to address barriers and leverage facilitators to PA. Proposed interventions targeted four selected areas of the neighbourhood and included expanding shaded areas, creating pedestrian-friendly routes and enhancing green spaces. This study highlights the effectiveness of a CoP in identifying and addressing barriers to PA within urban environments for people with chronic diseases. Findings emphasise the impact of neighbourhood design and accessibility on reducing SB and promoting active lifestyles. The participatory approach offers a replicable model for other urban settings aiming to foster health, although its qualitative and local nature limits generalisability.

## 1. Introduction

Urban environments play an important influence in influencing healthy lifestyles and reducing sedentary behaviour (SB), particularly as facilitators of physical activity (PA), a key factor of health. SB is a major risk factor for non-communicable diseases (NCDs), including cardiovascular disease, diabetes, cancer, and chronic respiratory conditions [1]. It also contributes to overweight and obesity, which in turn are independent risk factors for NCDs [1]. With the majority of the world’s population residing in urban areas, cities have the opportunity—and responsibility—to address these public health challenges [2]. The increasing prevalence of NCDs has made it essential to optimise urban spaces to support healthier lifestyles, particularly for people presenting chronic conditions [2]. Globally, 1 in 3 women and 1 in 4 men do not meet the recommended levels of PA, and it has been estimated that up to 5 million deaths a year could be prevented if populations were more active [3].

Urban infrastructure strongly influences people’s ability to incorporate PA into daily life. Well-designed environments that offer safe, accessible, and inclusive spaces can support regular activity, which is especially important for people with chronic diseases who may face mobility or health-related challenges [2,4]. Urban planning also shapes social and environmental determinants of health; features such as air pollution, noise, and unsafe traffic conditions can discourage PA and increase NCD risk, while investments in green areas, public transport, and pedestrian-friendly routes can help reduce these exposures and improve wellbeing [5,6]. As cities continue to grow, integrating health considerations into planning is increasingly relevant and aligns with global priorities such as the Sustainable Development Goals on health and sustainable cities [7]. Evidence from multiple contexts shows that walkable, connected, and green environments support active lifestyles, reduce environmental stressors, and help address health inequalities [8,9,10]. Studies across Europe indicate that access to green and recreational spaces is associated with higher levels of PA, lower obesity risk, and improved mental wellbeing, including among older adults and those with functional limitations [11,12,13,14]. These findings highlight the importance of inclusive and health-oriented urban design in reducing barriers and promoting PA across diverse populations.

In this context, a Community of Practice (CoP) could be a valuable strategy for developing and implementing proposals to reduce SB, enhancing urban environments and promoting PA for people with chronic diseases [15]. A CoP is defined as “groups of people who share a concern, a set of problems, or a passion about a topic, and who deepen their knowledge and expertise by interacting on an ongoing basis” [16]. This approach enables the co-creation of solutions and encourages the integration of local insights with scientific evidence, ensuring that the strategies do not only sound in theory, but are feasible in practice. By facilitating dialogue and collaboration, a CoP can ensure that interventions are adaptable and sustainable, therefore leading to more equitable and effective approaches [15,16]. A CoP-driven learning approach has the potential to eliminate interprofessional barriers shaped by historical hierarchies and tensions [15,16]. Previous CoP implementations in diverse settings, including residential care and clinical rehabilitation, have shown success in translating evidence into practice and fostering collaboration across sectors [17,18,19,20]. These examples underscore the potential of CoPs to address urban health challenges, particularly in enhancing PA among populations with NCDs. This methodology allows for the collection of both quantitative and qualitative data, providing a comprehensive evaluation of proposals and strategies from multiple perspectives [21]. It covers not only the scientific aspects but also addresses the practical needs of the populations it aims to serve, ensuring that interventions are more effective and aligned with community needs [21].

La Florida is a densely populated neighbourhood located in L’Hospitalet de Llobregat within the Barcelona Metropolitan Area, Spain. It is a highly urbanised area, characterised by a mix of high-rise apartment buildings, narrow streets, and a limited number of public and green spaces [22]. The neighbourhood is home to a diverse and multicultural population, with a significant proportion of older adults and families with children. The socio-economic profile of La Florida is marked by high levels of unemployment, low income, and limited access to health-promoting resources such as recreational spaces and pedestrian-friendly infrastructure. These characteristics make La Florida a relevant case for studying the relationship between urban environments and public health, particularly in terms of promoting PA and addressing barriers related to the built environment. The latest Health Survey of Catalonia (2024) indicated that 40.9% of the total population in the South Barcelona Metropolitan Area (where La Florida is located) reported having a self-perceived chronic disease [23]. In addition, recent healthcare statistics from the Catalan Health System provide further insight into the health profile of La Florida [24]. Between 2017 and 2024, approximately 30% of the adult population (>15 years) attended primary care services for overweight or obesity, 23% for lipid metabolic disorders, and nearly 10% for type 2 diabetes. Also, between 14 and 23% of the populations reported a perceived poor health even without a diagnosed condition. Mortality data for the same period show rates of 20–25 deaths per 100,000 inhabitants due to endocrine, nutritional, and metabolic diseases; 185–215 from cardiovascular diseases; and 50–109 from respiratory diseases [24].

PA behaviour is shaped by interactions between individual, social, and environmental factors, which emphasises that capability, opportunity, and motivation must align for behaviour to occur [25]. These models are particularly relevant in disadvantaged neighbourhoods, where opportunities for outdoor activity are restricted by structural barriers. Integrating environmental-justice perspectives is also essential, as inequities in access to green spaces, safe infrastructure, and clean environments disproportionately affect low-income and multi-ethnic communities [10]. These frameworks collectively justify the need for participatory approaches that simultaneously address behavioural, social, and environmental determinants of PA. While CoPs have been successfully implemented in healthcare, rehabilitation, and professional learning settings, their application in urban planning and community health remains limited. Existing research shows that CoPs can improve collaboration and translate evidence into practice [17,19,21]. However, few studies have integrated residents with NCDs, urban planners, community organisations, and health professionals in a single participatory process to address built-environment determinants of active living.

As mentioned, there is limited evidence of the use of CoPs to co-design urban environment interventions specifically targeted at people living with chronic diseases in socioeconomically disadvantaged neighbourhoods. Existing CoP studies typically focus either on professional learning within health services or on technical collaboration among planners, without fully integrating residents with NCDs, frontline health professionals, municipal actors, and urban planners in a shared decision-making process [17,21]. Moreover, few participatory approaches explicitly address neighbourhoods affected by structural health inequities and environmental injustice—contexts where barriers to PA accumulate due to dense built form, limited green infrastructure, and socioeconomic vulnerability [10,26].

People living with chronic diseases face additional and distinct challenges to engaging in PA, including pain, reduced mobility, treatment side-effects, fatigue, fear of exacerbating symptoms, and psychological barriers such as low confidence or anxiety [27,28,29]. These challenges often intersect with environmental constraints in deprived neighbourhoods, creating compounded barriers to active living. A tailored participatory approach is therefore required to ensure that proposed urban interventions are feasible, acceptable, and responsive to the needs of this population. Recent work has highlighted how neighbourhood walkability, access to green areas, and proximity to recreational spaces influence PA behaviours, particularly during periods of restricted mobility such as the COVID-19 pandemic [30,31]. Proximity-based planning initiatives, such as the 15 min parks approach, show how spatial accessibility can enhance wellbeing and encourage active lifestyles in dense urban environments [30,31]. Incorporating this evidence strengthens the rationale for examining how residents and stakeholders can collaboratively identify place-based solutions within their own neighbourhoods.

The +ACTIU project addresses this gap by operationalising a CoP as a cross-sector mechanism for re-thinking the urban environment through the lens of chronic disease management. The novelty lies in (i) integrating lived experience of NCDs into urban design decision-making, (ii) bridging health and planning sectors in a highly disadvantaged Spanish urban area, and (iii) applying a structured CoP framework combined with an evidence-based urban health tool to generate actionable environmental proposals. In doing so, the study aims at contributing new insights into how participatory knowledge-sharing processes can support inclusive urban health strategies for chronic disease populations. The primary aim of the +ACTIU project was to collaboratively develop strategies through a CoP, to reduce SB and promote PA in La Florida urban setting, with a particular focus on people with NCDs.

## 2. Materials and Methods

### 2.1. Methods

This study approaches a community-based participatory research [32], informed by principles of participatory action research [33]. Participatory approaches are particularly valuable when addressing complex, place-based determinants of health, as they recognise that residents, practitioners, and local institutions hold complementary forms of knowledge that are essential for designing feasible and context-specific interventions [32]. Within this model, the +ACTIU project is based on co-creation, collective learning, and multidisciplinary knowledge exchange. This methodology integrates shared decision-making and reflection, as well as the integration of the lived experience with professional and technical expertise. Table 1 summarises the stages of participatory research [34] and illustrates how they were implemented in this project.

### 2.2. Study Design

The +ACTIU project (www.mesactiu.com) is a multi-phase participatory research study aimed at enhancing urban environments to reduce SB and promote PA, with a specific focus on people with chronic diseases. Its area of focus is La Florida neighbourhood in L’Hospitalet de Llobregat, within Barcelona Metropolitan Area. La Florida was selected as the study setting due to its combination of high population density, socioeconomic vulnerability, and limited access to quality public spaces. Previous municipal reports have highlighted local concerns related to mobility, green-space scarcity, and chronic disease burden in this neighbourhood. These factors make La Florida a relevant context for testing a participatory, cross-sector approach aimed at improving opportunities for PA [35].

While the broader project encompasses multiple phases, this paper specifically focuses on the initial stages. Here, we detail the methodology and results of the first phase of the project, which include the development of a CoP for collaborative strategy formulation. These phases set the stage for subsequent interventions and evaluations that will be addressed in future work.

### 2.3. Community of Practice Implementation

**Figure 1:**
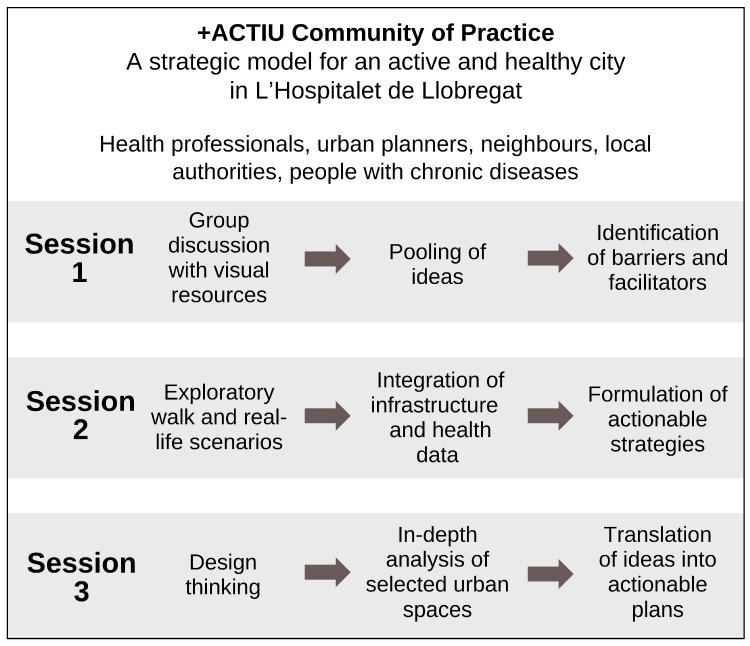
Overview of the methodology employed in the +ACTIU Community of Practice.

The +ACTIU CoP consisted of a participative and interactive discussion with dynamic groups that shared interests and learnt to improve, by co-designing innovative solutions, certain problems identified in the urban environment of La Florida. This methodology was established in order to achieve impactful solutions through multidimensional and transdisciplinary points of view, inspired by responsible research and innovation, promoting the participation of a variety of people and organisations. Three sessions were organised, where representatives of the community, health professionals, urban planners, innovation and education professionals, local authorities, as well as people from the general population with NCDs, gathered to develop strategies, analysed the situation and co-developed strategies for future implementation. Ethical approval for this project was granted by the Ethics Committee at Bellvitge University Hospital. To ensure broad and equitable participation, recruitment followed a targeted, multi-channel strategy. In addition to email invitations, we conducted active outreach through neighbourhood libraries, civic centres, community health services, municipal facilities, and local associations supporting people with chronic diseases. Personalised phone calls and in-person invitations were used to reach residents who are typically under-represented in participatory initiatives. Inclusion criteria were: (i) living, working, or providing services in La Florida; (ii) being ≥18 years old; and (iii) belonging to one of the stakeholder groups defined for the CoP (community residents, people with NCDs, health professionals, urban planners, or local authorities). No exclusion criteria were applied beyond inability to provide informed consent. All workshops were audio-recorded with and complemented by detailed field notes taken independently by two researchers. Visual materials generated during the sessions (photographs, maps, group worksheets, sticky-note clusters, and prioritisation matrices) were collected and digitised. For the Healthy Cities Generator session, output files and screenshots of scenario analyses were also archived. This multimodal documentation ensured a comprehensive and transparent record of the CoP process. All participants signed informed consent and image release forms to participate. Figure 1 presents an overview of the methodology employed in the CoP.

The first session of the +ACTIU CoP focused on identifying barriers and facilitators (B&F) to PA within the urban environment of L’Hospitalet de Llobregat, specifically the Florida-Les Planes area. It was held in the Módulo from the Contorno Urbano Foundation in L’Hospitalet de Llobregat, a community-driven ecological and cultural centre that promotes urban regeneration through participatory design, sustainable construction, and cultural programmes for local engagement and social inclusion [24]. This session aimed at collecting insights to develop strategies for promoting PA in the urban setting. Researchers selected different images related to B&F of PA based on previous literature [36]. Participants were guided through group discussions, which generated valuable qualitative data on the lived experiences of residents and professionals in relation to urban design and health promotion.

The second session consisted of a comprehensive evaluation of the urban environment in the Florida-Les Planes area. The Healthy Cities Generator (HCG) was used: an evidence-based digital tool to assess urban health determinants to incorporate health factors into urban planning and urban factors into health policy [37]. Held at the Municipal Office of the Florida-Les Planes Integrated Planning [35], the session aimed to identify key urban factors influencing active lifestyles and to prioritise interventions that address previously identified barriers. Analysing through five real-life scenarios, participants explored the study area, assessing determinants such as accessibility for older adults, safe commuting routes, and spaces conducive to community-based physical activities. During this diagnostic process, participants collectively identified several points along the avenue where recurrent issues converged—such as poor walkability, insufficient greenery, lack of shade, environmental nuisances (noise, air pollution), or deteriorated public infrastructure—as well as areas with strategic potential due to their proximity to parks, civic centres, or daily services. Using the HCG tool [37], these observations were synthesised and integrated into a prioritisation matrix, from which four representative “places of opportunity” were selected to reflect different spatial typologies and feasible scales of intervention within the neighbourhood. These predefined locations then served as the basis for the design-thinking activities in the third session, guiding the co-design of actionable strategies aimed at improving the urban environment and enhancing opportunities for PA.

**Figure 2:**
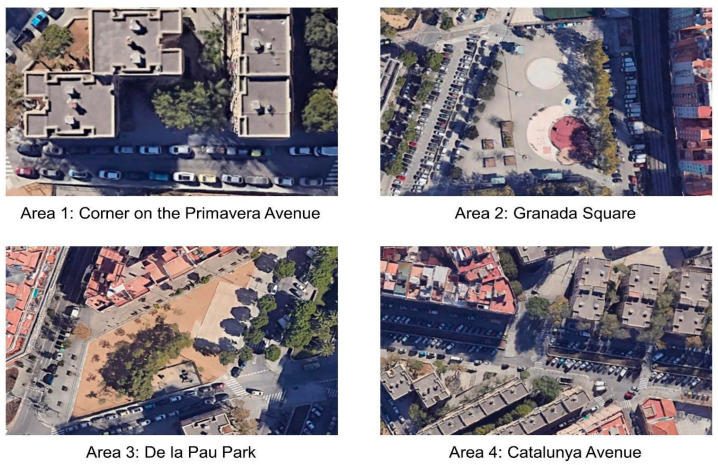
Urban areas of session 3. Source: Google Maps Satellite View © Google 2025 [39].

The third session focused on defining priority actions to address the needs identified during the previous workshops. It was held at the Ana Díaz Rico Municipal Centre, a community-oriented civic and cultural public facility that promotes social cohesion and local engagement through a variety of educational, cultural, and recreational activities, centrally located in the Florida—Les Planes area. We used design thinking [38] to translate ideas into actionable plans for pre-selected spaces along the avenue. Places of opportunity of different scales were identified, with the objective that the solutions devised could then be easily replicated in other contexts. Figure 2 shows the four areas selected for the proposals, based on the results of the previous workshop. The collaborative process aimed at developing robust, holistic proposals to improve the urban environment. These outcomes laid the foundation for the next stage of the project: implementing a pilot test to trial these interventions.

To ensure the trustworthiness of the qualitative findings, methodological triangulation was achieved by combining recordings, researcher observations, and visual outputs. In addition, member checking occurred informally during the workshops as preliminary interpretations were discussed and refined with participants. Reflexive notes were maintained throughout the process to document researcher assumptions and enhance transparency.

## 3. Results

Table 2 summarises the composition of participants across three sessions. The number of participants in each session ranged from 24 to 25. Participation was limited to two gender identities: men and women, with most being women (64–71%). People from the general population with chronic diseases accounted for 48%, 32%, and 25% of participants in Sessions 1, 2, and 3, respectively. Health professionals formed another key group, contributing 40–48% of participants. Other categories, such as urban planners, students, researchers, and public officials, had variable representation, with women predominating in most groups.

Session 1 focused on identifying the personal and environmental B&F that impact PA within the urban environment of L’Hospitalet de Llobregat. The qualitative analysis revealed that B&F could be divided into two main categories: personal and environmental. Table 3 presents a categorisation of the B&F for PA identified during Session 1 of the CoP.

Personal barriers included physical limitations such as a lack of mobility, chronic pain, and reduced physical condition, as well as psychological factors like boredom, depression, and low motivation. Time constraints due to work and family responsibilities were also highlighted as significant social barriers to PA engagement. On the other hand, personal facilitators included motivations such as health promotion, increased energy levels, and the enjoyment of exercise. Social factors, including support from family and peers, were identified as key facilitators that encouraged PA.

Environmental barriers were primarily related to spatial and infrastructure limitations, such as heavy traffic, narrow sidewalks, and a lack of safe green spaces. Participants also cited poor air quality and extreme weather conditions as factors that limited their ability to engage in outdoor PA. In contrast, environmental facilitators included access to well-maintained green spaces, pedestrian-friendly areas, and favourable weather conditions, which motivated participants to partake in outdoor PA. These findings highlight the complex interplay between personal and environmental factors in shaping PA behaviour, suggesting that any intervention aimed at promoting PA must address both personal and environmental barriers while taking advantage of facilitators effectively.

**Figure 3:**
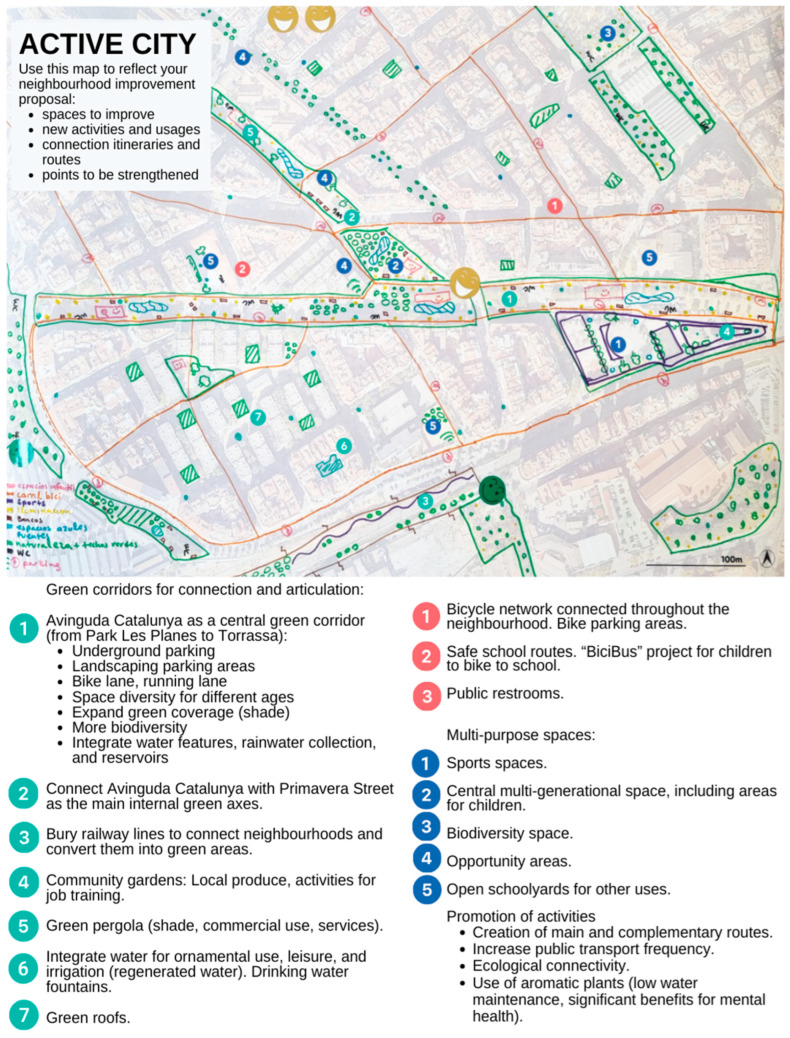
Urban improvement activity and proposals for physical activity promotion: outcomes from Session 2 of the +ACTIU Community of Practice. Source: Google Maps Satellite View © Google 2025 [39].

During the second session of the CoP, participants carried out a detailed assessment of the Florida-Les Planes area and its potential to support active lifestyles. They systematically identified B&F to PA within the neighbourhood. Among the key facilitators observed were the existing green spaces, particularly Les Planes Park, which serves as a central hub for various forms of exercise and community interaction. The park’s availability of exercise equipment, playgrounds, and shaded areas was highlighted as a significant asset in encouraging PA. Additionally, the accessibility and connectivity of services within the area, such as local markets, civic centres, and healthcare facilities, were perceived as beneficial factors that promote active transportation, such as walking and cycling. The presence of wide sidewalks and pedestrian zones in certain parts of the neighbourhood, combined with adequate street lighting during the day, also contributed to a sense of safety and encouraged the use of these spaces. However, several barriers were also identified, such as the limited number of green and recreational spaces beyond Les Planes Park. Many participants noted that the neighbourhood’s public spaces were overly paved, with insufficient greenery, which reduced their appeal for PA. Environmental factors, including high levels of air and noise pollution from vehicular traffic and nearby railway tracks, were seen as significant disincentives, particularly in areas lacking shade and water fountains. Additionally, the poor condition of some public infrastructure, such as narrow and uneven sidewalks, and unlit pathways, further hindered active mobility. Social and structural barriers were also noted, such as empty storefronts and unused lots, which created a feeling of abandonment and made the area less appealing for outdoor activities. The high density of buildings and overcrowded spaces also contributed to a less inviting atmosphere, reducing opportunities for PA. Following these observations, participants engaged in a collaborative diagnosis workshop to synthesise their findings and develop targeted strategies to improve the neighbourhood’s urban environment. Using the HCG tool, they prioritised interventions such as creating continuous green corridors to connect existing parks and open spaces and enhancing pedestrian accessibility through infrastructural improvements. Participants also proposed the revitalisation of neglected areas, suggesting the transformation of abandoned lots into community gardens and recreational spaces. The session concluded with the formulation of actionable strategies aimed at fostering an active and healthy urban environment. Figure 3 summarises these actions.

In Session 3 of the +ACTIU CoP, participants focused on developing targeted proposals to transform selected areas within the Florida-Les Planes neighbourhood to better support PA and social engagement for people with chronic diseases. Four teams assessed potential improvements in specific locations (see Figure 2). Proposals emphasised creating multifunctional spaces to foster intergenerational engagement and support diverse activities, from structured exercise circuits to informal gatherings. Table 4 summarises the main findings and proposals from this session, highlighting how each site is tailored to the specific needs of the community while also suggesting strategies for a more inclusive and engaging urban environment.

## 4. Discussion

Our findings underscore the important role of urban design in promoting PA and supporting health among populations with chronic diseases. The participatory, community-driven approach of the CoP facilitated the identification of both physical and social elements within the Florida-Les Planes neighbourhood that influence active lifestyles. Through the collaborative workshops, participants highlighted the importance of accessible, adaptable, and interconnected urban spaces to address barriers and leverage facilitators to PA. The proposed interventions—such as expanding shaded areas, creating pedestrian-friendly routes, and enhancing green spaces in the form of, for example, community gardens—reflect a holistic understanding of the community’s needs and a shared commitment to creating a supportive environment for PA.

The findings of this study advance theoretical understanding of how participatory mechanisms can support health-promoting urban transformations in disadvantaged settings. By positioning chronic disease populations as active contributors rather than passive beneficiaries the +ACTIU CoP illustrates how collective learning can bridge sectors that are often separate in practice (public health, clinical care, municipal planning, and community organisations). This aligns with socio-ecological and environmental-justice frameworks, which emphasise the interdependence between individual capabilities, social conditions, and structural features of the built environment [40]. Our results extend these frameworks by demonstrating how CoPs can function as a practical tool to operationalise them: enabling multidirectional knowledge exchange, identifying inequitable exposure to environmental barriers, and collectively generating feasible spatial proposals. From a theoretical perspective, the study contributes a model for integrating CoP methodology into urban health interventions aimed at NCD populations, showing how experiential knowledge (residents with chronic diseases), professional expertise (healthcare professionals), and spatial-technical insights (urban planners) can be synthesised to inform inclusive, place-based strategies. This cross-sector integration represents an important step forward in conceptualising how collaborative governance mechanisms can address inequities in PA opportunities in dense, low-resource urban environments.

In Session 1, we identified several physical and psychological barriers to PA. These included limited mobility, pain or discomfort, boredom, low mood, and a perceived poor health condition. These findings align with a recent qualitative study [27] on participants with chronic diseases, which highlighted lack of self-confidence as a significant obstacle to PA, alongside functional limitations and feelings of embarrassment about low physical fitness. Within medical practice, people often avoid discussing these challenges, despite a desire to be more active [27]. Additionally, certain treatments, particularly those related to cancer, are associated with side effects such as fatigue, pain, muscle weakness, and nausea, further limiting PA [28]. Conversely, a key psychological facilitator for PA in our study was awareness of its health benefits. Participants often link motivation not only to a general understanding of PA’s positive impact on health but also to its role in managing specific chronic conditions. Enjoyment, personal satisfaction, and opportunities for social interaction emerged in our study as major facilitators of PA. As for social factors, lack of time and insufficient social support were the primary barriers, whereas encouragement from family and friends significantly facilitated participation in PA. These observations are consistent with existing literature [28,29], emphasizing the importance of a supportive social environment in promoting sustained PA.

Certain behaviours are more likely to occur when ability, motivation, and environmental opportunities align, and motivation increases with ability and supportive environments [28,29]. After assessing environmental factors that hinder or support PA, we observed that traffic, tight sidewalks, lack of green spaces, air pollution, and meteorological conditions were the greatest barriers of PA. Conversely, access to areas with green space, adapted to pedestrians, and protected from weather conditions were major facilitators of PA. A recent Spanish study observed that the perception of having nearby green spaces for walking, movement, or leisure was linked to greater adherence to PA, especially when these areas were close to home [41]. Likewise, living in areas with greater accessibility to green spaces increased the probability of meeting WHO’s PA recommendations by 24% compared to areas with lower accessibility in an English population [11]. A recent study across eight countries in Europe, South America, and Asia used a photovoice approach to explore perceptions of PA determinants in public spaces [42]. Safe walking infrastructure was a universal concern but varied by context: transport safety in Europe, space competition with vendors in Nepal and Hong Kong, crime in Brazil, and accessibility for disabled people in India. These findings highlight the shared importance of walking infrastructure, tailored to local challenges [42].

In line with these findings, international evidence reinforces the importance of high-quality green and natural spaces as determinants of both physical and mental well-being [13,43]. Regular exposure to natural environments has been linked to reduced stress, improved emotional regulation, and increased motivation to engage in PA, particularly among populations with chronic diseases or functional limitations. Recent studies highlight that not only the presence of green areas but also their perceived quality, safety, and accessibility influence activity levels and mental health benefits, underscoring the need for well-maintained, inclusive, and multifunctional public spaces [43]. Contact with greenery through urban trees, parks, community gardens, or small-scale nature-based solutions contributes to greater psychological restoration and enhances the overall liveability of dense urban neighbourhoods, especially in socioeconomically disadvantaged areas [43]. These insights reinforce the need for intersectoral collaboration between public health, urban planning, and environmental services to ensure that investments in green infrastructure also support long-term community wellbeing and health equity.

The CoP approach has proven effective in various contexts, demonstrating its versatility and impact in addressing urban health challenges. By bringing together diverse stakeholders—community members, health professionals, urban planners, and local authorities—CoPs facilitate the co-creation of solutions tailored to local needs to help bridge the health equity gap by making PA opportunities more accessible to all residents [44]. Previous studies have shown the success of CoPs in translating evidence into practice, particularly in healthcare settings, residential care, and clinical rehabilitation [17,18,19,20]. This participatory framework is especially relevant for urban spaces, where the intersection of built environments, social dynamics, and health behaviours requires multidimensional strategies. By integrating local knowledge and perspectives with scientific evidence, the +ACTIU CoP enabled the design of urban interventions that are practical, inclusive, and sustainable. These interventions addressed barriers such as accessibility, safety, and inclusivity, while considering health equity. It could be considered a starting point for future collaborative action and policy making encompassing urban settings and health in the Metropolitan Area of Barcelona, serving as a model for similar initiatives in other urban contexts.

This study has limitations that should be considered. While this approach could serve as a foundation for similar initiatives, its applicability may be limited by local cultural, social and environmental differences. The qualitative nature of the data introduces a level of subjectivity; however, we employed evidence-based methodologies [36,38] and involved diverse stakeholders to ensure a comprehensive perspective. The cross-sectional design provides only a snapshot of the current situation, which may not capture long-term trends or the sustainability of proposed interventions. However, the participatory approach of the CoP helps keep stakeholders involved over time, allowing for regular reviews and adjustments to the interventions. The implementation of the identified strategies could face potential barriers, such as securing political will, adequate funding, resource availability, and sustained community engagement, all of which are critical for the success of urban health initiatives. The demographic information collected from participants included self-identified gender. In our sample, all participants identified as either women or men, and no participants reported another gender identity. We acknowledge this as a limitation of the sample rather than of the data collection instrument. Importantly, we did not purposively seek participants of specific gender identities to avoid introducing selection bias. As a result, gender diversity may not be fully represented in this CoP. These limitations will be addressed in the next phase of the +ACTIU project. The development of a pilot intervention study is planned to implement and evaluate the proposed strategies in a real-world setting, allowing for a more comprehensive assessment.

Although the CoP methodology promotes collaboration across diverse stakeholders, we recognise that participatory environments can still be affected by differences in confidence, professional authority, or perceived expertise. To mitigate these dynamics, participants were intentionally assigned to heterogeneous groups that mixed residents with chronic diseases, community members, health professionals, urban planners, and municipal actors. This structure avoided clustering people by profession or status. Each group was guided by a moderator who actively facilitated discussion, encouraged contributions from quieter participants, and ensured that no single voice or professional profile dominated the conversation. While these measures helped foster balanced participation, we acknowledge that subtle power dynamics may still have influenced interactions, as is common in multi-sector participatory processes.

Finally, the long-term impact of the CoP depends on steps that take place beyond the participatory sessions described here. The proposals generated by the CoP were conceived as inputs for subsequent phases of the +ACTIU project, where they will be reviewed, refined, and prioritised with institutional stakeholders. In this sense, the CoP functions as an initial stage within a broader translational process aimed at informing municipal decision-making and supporting the integration of community-driven recommendations into local policy and planning agendas. The effectiveness and sustainability of the proposed interventions will ultimately rely on these next steps, including the engagement of governmental, technical, and professional actors responsible for implementation and long-term maintenance in the urban environment.

This study has several strengths that enhance its relevance and potential impact. The participatory approach, through the CoP, ensured the active involvement of diverse stakeholders, including community members, health professionals, urban planners, policymakers, and people with chronic diseases, fostering co-created and locally tailored strategies. The use of the HCG tool [37] provided a structured and dynamic method to assess urban health determinants and prioritise interventions effectively. A key strength was the focus on vulnerable populations, particularly people with chronic diseases living in L’Hospitalet de Llobregat, ensuring that the proposed strategies addressed the needs of those highly affected by barriers to PA. Finally, the study’s emphasis on actionable outcomes offers practical solutions that can be directly implemented to improve urban environments and promote health.

## 5. Conclusions

This study highlights the effectiveness of a CoP in identifying and addressing barriers and facilitators to active lifestyle within urban environments, particularly for people with chronic diseases. Findings emphasise the impact of neighbourhood design and accessibility on promoting PA and reducing SB. By engaging residents and authorities, health experts and urban planners, the CoP generated practical evidence-based recommendations to improve the Florida-Les Planes area, such as enhancing accessibility, increasing greenery, and revitalising public spaces. Although the study’s scope is limited to this specific area, the participatory approach offers a replicable model for other urban settings aiming to reduce SB and foster an active lifestyle.

Future research should examine the implementation and long-term effects of the proposed interventions, assessing whether CoP-based strategies lead to sustained changes in PA and urban health. Theoretically, this study contributes to understanding how CoPs can operationalise equity-oriented urban health frameworks by integrating experiential, clinical, and spatial knowledge. Practically, the findings underscore the value of cross-sector collaboration and participatory planning to guide feasible, context-specific urban improvements, offering insights that can support policy development and municipal action in similar neighbourhoods.

## Figures and Tables

**Table 1:** Stages of participatory action research and their application in +ACTIU.

Stage	Description	Implementation in +ACTIU
1. Background	Get to know people in the community and what they do. Build trust.	Initial engagement with local authorities, community organisations, and health services. Identification of local concerns related to SB, B&F for PA, and NCDs. Establishment of trust and shared purpose before starting the CoP.
2. Agreement	People decide whether to join the research programme on the basis of mutual understanding and an agreed direction.	Recruitment of residents with NCDs, community members, health professionals, urban planners, and local authorities through a multi-channel strategy. Participants joined voluntarily after being informed of the aims, approach, and expected level of involvement.
3. Choosing the Questions	Follow community lead; communities know the issues that need researching.	Session 1: Participants jointly identified key B&F affecting active living and prioritised themes to explore in depth.
4. Research Methods and Data Collection	Agree how best to reach people and collect the data.	Session 2: Participants conducted a structured neighbourhood assessment using the HCG, scenario analysis, and group mapping. They generated observational, experiential, and spatial data on environmental determinants of PA.
5. Data Analysis	Pull all the responses together. Summarise community position.	Findings from Session 2 were synthesised collaboratively using prioritisation matrices and discussion. The research team and planners helped organise insights into four “places of opportunity.”
6. Key Findings	Write up the information. Extract the key findings.	Key environmental barriers and facilitators were summarised and grouped into thematic categories. These findings informed the areas targeted in Session 3.
7. Presentation	Share findings with all stakeholders. Reflect on what next.	Results from Session 2 were presented back to participants at the start of Session 3, enabling reflection and preparing the group for the co-design activities.
8. Action	Take action on the findings. Build on connections and experience gained.	Session 3: Groups used design-thinking to co-create actionable proposals for improving four selected areas. These outputs constitute the foundation for the upcoming +ACTIU pilot intervention.

B&F: barriers and facilitators; CoP: community of practice; HCG: healthy cities generator; NCDs: non-communicable diseases; PA: physical activity; SB: sedentary behaviour.

**Table 2:** Participant distribution by session from the +ACTIU Community of Practice.

	Session 1 (*n* = 25)	Session 2 (*n* = 25)	Session 3 (*n* = 24)
Gender (women), *n* (%)	16 (64)	17 (68)	17 (71)
Age (years), mean (SD)	50 (15)	51 (16)	50 (15)
Stakeholder profiles, *n* (%)			
People with NCDs ^1^	12 (48)	8 (32)	6 (25)
Overweight/Obesity	6 (50)	5 (62.5)	4 (66)
Hypertension	4 (33)	3 (37.5)	2 (33)
MSK/Chronic pain	4 (33)	3 (37.5)	2 (33)
Other ^2^	3 (25)	3 (37.5)	2 (33)
Health professionals	10 (40)	12 (48)	11 (46)
Urban planners	2 (8)	3 (12)	5 (21)
Students	1 (4)	1 (4)	1 (4)
Public officials	-	1 (4)	1 (4)

MSK: musculoskeletal condition; NCDs: non-communicable diseases. ^1^ Category percentages use the NCD subgroup as denominator; the total NCD percentage uses the full sample; values exceed 100% because some participants reported multiple conditions. ^2^ Other NCDs: cancer, chronic obstructive pulmonary disease, coronary heart disease, type 2 diabetes.

**Table 3:** Barriers and facilitators for PA: insights from Session 1 of the +ACTIU Community of Practice.

	Personal Factors			
	Physical	Psychological		Social
Barriers	Intensity of activities: The level of effort required was perceived as too high, especially for people not used to practicing regular exercise. Lack of mobility: Disabilities, chronic illnesses, or reduced physical capacity were reported as major impediments. Pain and discomfort: Chronic or acute pain deterred regular participation in PA.	Boredom: The monotony during PA sessions reduced motivation. Depression and low mood: These factors led to decreased motivation and energy to participate in PA. Perceived poor health: Even without a diagnosed condition, the perception of poor health discouraged PA engagement.		Time constraints: Multiple obligations (work, family) limited time for PA. Lack of social support: Some participants felt discouraged from engaging in PA due to lack of company or social encouragement.
Facilitators	Prevention and promotion of health: participants acknowledged that engaging in PA could prevent diseases and promote overall well-being. Increased energy levels: Participants reported feeling more energetic and vital when engaging in PA.	Enjoyment and personal satisfaction: participants reported that finding PA enjoyable made it easier to maintain a routine. Socialisation opportunities: PA offered opportunities to interact with others, which encouraged participation.		Support from family and peers: having social support and participating in different group activities.
	Environmental Factors			
	Spatial and infrastructure		Environmental conditions	
Barriers	Heavy traffic and narrow sidewalks: These were perceived as dangerous and discouraged walking or outdoor activities. Lack of green spaces and parks: A dearth of attractive, safe public spaces was reported as a major impediment. Pollution: Air quality was identified as a significant barrier to engaging in outdoor activities.		Extreme weather: High temperatures or heavy rain made outdoor PA unappealing and, at times, unsafe.	
Facilitators	Access to green spaces: participants highlighted that the presence of parks and green areas was a major incentive for outdoor PA. Availability of pedestrian-friendly areas: Proper sidewalks and walking routes facilitated regular PA.		Good weather conditions: Favourable weather was seen as a major enabler for engaging in outdoor PA.	
PA: physical activity.				

**Table 4:** Proposed strategies from Session 3 of the +ACTIU Community of Practice.

Area 1: Corner on the Primavera Avenue		
Proposed uses & activities	Target population	Physical interventions
Meeting and gathering place. Bike parking.	Intergenerational. Children.	Installation of pergola-type structure Options: Square with shade, lighting, bench-type base and chess tables, pedals, etc. Cyclist square with bike parking and repair station Goose square with a goose game on the ground for physical activity.
Area 2: Granada Square		
Proposed uses & activities	Target population	Physical interventions
Playground Gym Basketball court Vegetable Garden area (training activities) Petanque area	Intergenerational. Population at risk of exclusion.	Reduction in parking to improve accessibility. Integrated parking, change of pavement. Connection with cycle lane Differentiation of traffic lanes (and speeds) by different colours. Installation of toilets and information booth. Playground area with natural elements, sand. Water (sprinklers) Grass (natural or artificial) for the ‘gym’ area. Shade element easy to install.
Area 3: De la Pau Park		
Proposed uses & activities	Target population	Physical interventions
Seating and exercise area. Directed activities by levels, integrated in a circuit.	Intergenerational. Population with functional diversity.	Open a perimeter wall for ramped access. Installations of fountains, and toilets. Use of bio-healthy machines, pedals, etc. Exercise lanes for people with functional diversity, walking paths.
Area 4: Catalunya Avenue		
Proposed uses & activities	Target population	Physical interventions
Socialization through vegetable gardens. Zone with water access. Exercise throughout the Avenue by means of a circuit.	Intergenerational. Associations.	Creation of vegetable gardens. Installation of exercise structures: circuits combining stairs and stations with different structures. Bicycle parking. Closed water circuit in the centre of the park. Installation of structures to favour shades.

## Data Availability

The data supporting the findings of this study are available upon reasonable request from the corresponding author. The data are not publicly available as they contain information that could compromise the privacy of the study participants.

## Abbreviations

The following abbreviations are used in this manuscript:

B&F	Barriers and facilitators
CoP	Community of practice
HCG	Healthy cities generator
NCD	Non-communicable diseases
PA	Physical activity
SB	Sedentary behaviour
